# Supplementary material for: Genomic Characterization of Escherichia coli Isolates Belonging to a New Hybrid aEPEC/ExPEC Pathotype O153:H10-A-ST10 eae-beta1 Occurred in Meat, Poultry, Wildlife and Human Diarrheagenic Samples
Source: Antibiotics (Basel). 2020 Apr 17;9(4):192. doi: 10.3390/antibiotics9040192 (PMC7235894; doi:10.3390/antibiotics9040192)
Supplement: Supplementary file 1 [file antibiotics-09-00192-s001.pdf]

Supplementary data

Genomic characterization of *Escherichia coli* isolates belonging to a new hybrid aEPEC/ExPEC pathotype O153:H10-A-ST10 *eae*-beta1 occurred in meat, poultry, wildlife and human diarrheagenic samples

Dafne Díaz-Jiménez [1+], Isidro García-Meniño [1+,] Alexandra Herrera [1], Vanesa García [1,4], Ana María López-Beceiro [2], María Pilar Alonso [3], Jorge Blanco [1], and Azucena Mora [1,*]

Table S1. Thirty-two isolates included in the study (in red) from our own collections

Origin of isolation	Sampling period	No. ESBL aEPEC O153 isolates / total ESBL isolates [a]	No. NON-ESBL aEPEC O153 isolates[b]
Chicken meat study	2009-2010	**7** / 127	NA
Beef meat 1st study	2005-2009	**5** / DNA	**2**
Beef meat 2nd study	2011-2012	**1** / 5	NA
Pork meat study	2011-2012	**1** / 13	NA
Poultry farm environment	2010-2012	**1** / 96	NA
Wildlife study	2014-2015	**1** / 95	NA
Human diarrhea	2006-2012	**5** / DNA	**9**

[a] Data not available (DNA); [b] Not analyzed (NA)

Table S2. Assembly data from Enterobase of the 17 O153:H10-A-ST10 genomes sequenced using Illumina NextSeq technology

Code	Assembly barcode	Coverage	N50	Length	Contig No. (>=200 bp)	ST_ 7 gene	ST Complex	wgMLST 25,002 loci	cgMLST 2,513 loci	rST 53 loci	O antigen prediction	H antigen prediction
LREC-110	ESC_KA7423AA_AS	361	147271	5152970	178	10	ST10 Cplx	38372	37600	2021	O153	H10
LREC-111	ESC_KA7425AA_AS	370	126323	5239837	221	10	ST10 Cplx	38373	37601	2021	O153	H10
LREC-112	ESC_KA7429AA_AS	124	109355	5084929	342	10	ST10 Cplx	38377	37605	2021	O153	H10
LREC-113	ESC_KA7430AA_AS	92	93205	5172711	213	10	ST10 Cplx	38378	37606	2021	O153	H10
LREC-114	ESC_KA7438AA_AS	163	126323	5201046	213	10	ST10 Cplx	38386	37614	2021	O153	H10
LREC-115	ESC_KA7437AA_AS	141	126291	5232022	228	10	ST10 Cplx	38385	37613	2021	O153	H10
LREC-116	ESC_KA7436AA_AS	118	124442	5187480	212	10	ST10 Cplx	38384	37612	2021	O153	H10
LREC-117	ESC_KA7433AA_AS	163	124771	5160744	169	10	ST10 Cplx	38381	37609	2021	O153	H10
LREC-118	ESC_KA7706AA_AS	39	69529	5166783	292	10	ST10 Cplx	39187	38299	2021	O153	H10
LREC-119	ESC_KA7435AA_AS	296	125664	4994631	189	10	ST10 Cplx	38383	37611	2021	O153	H10
LREC-120	ESC_KA7432AA_AS	150	102481	5263192	230	10	ST10 Cplx	38379	37607	2021	-	H10
LREC-121	ESC_KA7434AA_AS	71	73833	5134535	170	10	ST10 Cplx	38382	37610	2021	-	H10
LREC-122	ESC_KA7440AA_AS	168	124771	5209684	223	10	ST10 Cplx	38388	37616	2021	O153	H10
LREC-123	ESC_KA7439AA_AS	78	123102	5208501	253	10	ST10 Cplx	38387	37615	2021	O153	H10
LREC-124	ESC_KA7441AA_AS	201	119599	5258246	171	10	ST10 Cplx	38389	37617	2021	O153	H10
LREC-125	ESC_KA7442AA_AS	166	119599	5274856	272	10	ST10 Cplx	38390	37618	2021	O153	H10
LREC-127	ESC_KA7426AA_AS	208	126318	5253322	213	10	ST10 Cplx	38374	37602	58738	O153	H10

Raw reads were uploaded and automatically assembled in Enterobase (https://enterobase.warwick.ac.uk/) using SPAdes Genome Assembler v3.5. with a threshold on contigs of minimum 200 nt. Subsequently, the *de novo* assembled contigs were MLST (7 gene ST, wgST, cgST and rST) and serotype predicted using Enterobase typing tools

Table S3. HierCC designations from Enterobase for the 17 Spanish collection and other 7 related genomes within each cluster group. SNPs of the core genomic regions

Name (Enterobase)	Source Details [a]	Collection Year [a]	Country [a]	O Antigen	H Antigen	ST	Lineage	fimH allele	cgMLST	HC0	HC2	HC5	HC10	HC20	HC50	HC100	HC200	HC400	SNPs [b]
110084	DNA	DNA	DNA	O5	H27	10	A	54	8886	8886	8886	8886	8886	8886	8886	8886	8886	8224	
166357	Human; Homo sapiens	2015	United Kingdom	O40	H10	10	A	54	21500	21500	21500	21500	21500	21500	21500	21500	8224	8224	
208917	Human; Homo sapiens	2016	United Kingdom	O40	H10	10	A	54	21361	21361	21361	21361	21361	21361	21361	21361	8839	8224	
853984	Homo sapiens; human	2019	United Kingdom	O153	H10	10	A	54	124093	124093	124093	124093	124093	124093	124093	37600	8224	8224	
866428	Homo sapiens; human	2019	United Kingdom	O153	H10	10	A	54	129194	129194	129194	129194	129194	129194	124093	37600	8224	8224	
AM_LREC-110	Chicken meat	2010	Spain	O153	H10	10	A	54	37600	37600	37600	37600	37600	37600	37600	37600	8224	8224	37
AM_LREC-111	Fox faeces	2015	Spain	O153	H10	10	A	54	37601	37601	37601	37601	37601	37601	37600	37600	8224	8224	61
AM_LREC-112	Human clinical faeces	2011	Spain	O153	H10	10	A	54	37605	37605	37605	37605	37605	37605	37600	37600	8224	8224	361
AM_LREC-113	Human clinical faeces	2007	Spain	O153	H10	10	A	54	**37606**	**37606**	**37606**	**37606**	**37606**	**37606**	37600	37600	8224	8224	0
AM_LREC-114	Beef meat	2008	Spain	O153	H10	10	A	54	37614	37614	37614	37614	37614	37600	37600	37600	8224	8224	20
AM_LREC-115	Chicken meat	2009	Spain	O153	H10	10	A	54	37613	37613	37613	37613	37613	37613	37600	37600	8224	8224	101
AM_LREC-116	Human clinical faeces	2006	Spain	O153	H10	10	A	54	37612	37612	37612	37612	37612	**37606**	37600	37600	8224	8224	22
AM_LREC-117	Beef meat	2007	Spain	O153	H10	10	A	54	37609	37609	37609	37609	37609	37609	37600	37600	8224	8224	36
AM_LREC-118	Chicken breast	2009	Spain	O153	H10	10	A	54	38299	38299	38299	38299	38299	37615	37600	37600	8224	8224	24
AM_LREC-119	Beef meat	2007	Spain	O153	H10	10	A	54	37611	37611	37611	37611	37611	**37606**	37600	37600	8224	8224	15
AM_LREC-120	Beef meat	2011	Spain	-	H10	10	A	54	37607	37607	37607	37607	37607	37607	37600	37600	8224	8224	537
AM_LREC-121	Human clinical faeces	2007	Spain	-	H10	10	A	54	37610	37610	37610	37610	37610	37610	37600	37600	8224	8224	51
AM_LREC-122	Pork meat	2011	Spain	O153	H10	10	A	54	37616	37616	37616	37616	37616	37615	37600	37600	8224	8224	28
AM_LREC-123	Chicken meat	2010	Spain	O153	H10	10	A	54	37615	37615	37615	37615	37615	37615	37600	37600	8224	8224	25
AM_LREC-124	Human clinical faeces	2007	Spain	O153	H10	10	A	54	37617	37617	37617	37617	37617	**37606**	37600	37600	8224	8224	31
AM_LREC-125	Beef meat	2008	Spain	O153	H10	10	A	54	37618	37618	37618	37618	37618	**37606**	37600	37600	8224	8224	21
AM_LREC-127	Poultry farm environment	2010	Spain	O153	H10	10	A	54	37602	37602	37602	37602	37602	37602	37600	37600	8224	8224	54
E89	Broiler; Liver	2015	Denmark	uncertain	H10	7003	A	54	36964	36964	36964	36964	36964	36964	36964	36964	8224	8224	
Escherichia coli 2312	DNA	DNA	DNA	O40	H10	10	A	54	8224	8224	8224	8224	8224	8224	8224	8224	8224	8224	

[a] Data not available (DNA); [b] Not analyzed (NA); [b] SNPs of the core genomic regions present in 90% of the 17 compared genomes of our collection and using LREC-113 as reference

Table S4. Number of human stool samples analyzed and positive for aEPEC O153

Year	Nº stool samples	No. of positive samples (%) for aEPEC O153	No. of positive samples (%) for O153:H10 *eae*-beta1 *fim*$_{AVMT78}$
2006	1,842	4 (0.22)	1 (0.05)
2007	2,095	11 (0.52)	8 (0.4)
2008	1,001	5 (0.50)	3 (0.3)
2009	550	0 (0)	0 (0)
2010	514	0 (0)	0 (0)
2011	1,207	2 (0.50)	1 (0.08)
2012	2314	1 (0.04)	1 (0.04)
Total	9,523	23 (0.14)	14 (0.15)

Table S5. Twenty-three aEPEC O153 human isolates recovered in the period 2006-2012

Isolate code	Year of isolation	Symptomatology	O153 Serogroup	H10 Antigen	*eae* gene	*eae* beta-1 intimin	*fimH*$_{AVMT78}$ gene
22250.06	2006	Diarrhea	+	-	+	+	-
37979. 06	2006	Diarrhea	+	+	+	-	-
41824. 06	2006	Diarrhea	+	-	+	-	-
45990. 06 (LREC 116)*	2006	Diarrhea	+	+	+	+	+
57646. 06	2007	Diarrhea	+	-	+	-	+
18396.07 (LREC 124)*	2007	Diarrhea	+	+	+	+	+
19979. 07 (LREC 113)*	2007	Diarrhea	+	+	+	+	+
30981. 07 (LREC 121)*	2007	Diarrhea	+	+	+	+	+
31952. 07	2007	Diarrhea	+	+	+	+	+
32182. 07	2007	Diarrhea	+	-	+	-	-
32651. 07	2007	Hemorrhagic gastroenteritis	+	+	+	+	+
32884. 07	2007	Diarrhea	+	+	+	+	+
34535. 07	2007	Acute gastroenteritis	+	+	+	+	+
39044. 07	2007	Acute gastroenteritis	+	+	+	+	+
65905/07	2007	Hemorrhagic colitis	+	-	+	-	+
110431.08	2008	Hemorrhagic colitis	+	-	+	+	-
2477.08	2008	Diarrhea	+	-	+	-	-
21011. 08	2008	Diarrhea	+	+	+	+	+
38506. 08	2008	Diarrhea	+	+	+	+	+
40237. 08	2008	Diarrhea	+	+	+	+	+
48633.11	2011	Diarrhea	+	-	+	-	-
9727.011 (LREC 112)*	2011	Hemorrhagic colitis	+	+	+	+	+
55515.12	2012	Diarrhea	+	+	+	+	+

(*) code of those strains which were WG sequenced

Table S6. *in silico* characterization of seven *E. coli* related genomes from Enterobase using CGE databases

Name	Serotype	Phylo group	CHType	ST	Plasmid content Inc group (pMLST)	Acquired resistances	Virulence genes
866428	O153:H10	A	11-54	10	**IncF (F2:A-:B-)**<br>IncX1<br>Col156	*aadA1; catA1; mdf(A); tet(A)*	*astA, eae, espA, espB, gad, iss, mchF, nleA, tir*
853984	O153:H10	A	11-54	10	**IncF (F2:A-:B-)**<br>IncX1<br>Col156	*aadA1; catA1; mdf(A); tet(A)*	*astA, eae, espA, espB, gad, iss, mchF, nleA, tir*
166357	O40:H10	A	11-54	10	**IncF (F2:A-:B-)**<br>IncHI2 (ST4)<br>IncQ<br>Col156	*$bla_{TEM-1B;}$ aph(3´´)-Ib, aph(3´)-Ia, aph(6)-Ib; catA1; mdf(A); florR; tet(A); sul2; dfrA8*	*astA, eae, espA, espB, gad, iss, mchF, nleA, nleC, tir*
E. coli 2312	O40:H10	A	11-54	10	**IncF (F2:A-:B-)**<br>IncI1 (STunknown)<br>Col156<br>Col (MG828)	*aac(3)-IV, aph(3´´)-Ib, aph(3´)-Ia, aph(4)-Ia, aph(6)-Ib; mdf(A); tet(A); sul2*	*astA, eae, espA, espB, gad, ireA, iss, mchF, nleA, nleC, tir*
E89	ND:H10	A	11-54	10	**IncF (F2:A-:B-)**<br>Col156	*aadA1; mdf(A);*	*astA, eae, espA, espB, gad, iss, mchF, nleA, tir*
208917	O40:H10	A	11-54	10	**IncF (F2:A-:B-)**<br>Col156	*mdf(A)*	*astA, eae, espA, gad, ireA, mchF, nleA, nleC, tir*
110084	O5:H27	A	11-54	10	IncF (F-:A-:B-)<br>pO111	*aph(6)-Ib; mdf(A); sul2; dfrA8*	*astA, celB, eae, espA, gad, iss, mchF, nleA, tir*

Table S7. Targets and primers associated with diarrheagenic pathotypes of *E. coli*

Pathotype	Target	Primers	Nucleotide sequence (5´- 3´)	Size (bp)	Reference
STEC	*stx1*	VT1-F	TCGCTGAATGTCATTCGCTCTGC	539	Mora et al., 2011
		VT1-R	TCAGCAGTCATTACATAAGAAC		
STEC	*stx2*	VT2-F1	TTTCTTCGGTATCCTATTCCC	358	Mora et al., 2011
		VT2-F2	TGTCTTCAGCATCTTATGCAG		
		VT2-R	CTGCTGTCCGTTGTCATGGAA		
EPEC	*eae*	EAE-V3F	CATTGATCAGGATTTTTCTGGT	510	Mora et al., 2011
		EAE-MBR	TCCAGAATAATATTGTTATTACG		
EPEC	*eae*	[a]EAE-R11	TCTTCGGAGGGTTTTTTATT	1125	Alonso et al., 2017
		[a]EAE-FBN	CAGGTCGTCGTGTCTGCTAAAAC		
EPEC	*eae*	[a]EAE-R12	CCAGACGAATATATACATATTC	1181	Alonso et al., 2017
		[a]EAE-FBN	CAGGTCGTCGTGTCTGCTAAAAC		
tEPEC	*bfp*	BFP-NF1	ATGGTTTCTAAAATCATGAATAAG	262	Bennett, 2003<br>García-Meniño et al., 2018
		BFP-NR1	ATTATTCCGGAATTGCAGATGTGT		
ETEC	*estA*	STa-A	ATTTTTATTTCTGTATTGTCTTT	176	Penteado et al., 2002
		STa-B	GGATTACAACACAGTTCACAGCAGT		
ETEC	*estB*	Stb-F	ATCGCATTTCTTCTTGCATC	175	Blanco et al., 1997
		Stb-R	GGGCGCCAAAGCATGCTCC		
ETEC	*eltA*	LT-A-1	GGCGACAGATTATACCGTGC	696	Schultsz et al., 1994
		LT-A-2	CCGAATTCTGTTATATATGTC		
EIEC	*ipaH*	EI1	GCTGGAAAAACTCAGTGCCT	424	Tornieporth et al., 1995
		EI2	CCAGTCCGTAAATTCATTCT		
EAEC	*aatA*	pCVD432/start	CTGGCGAAAGACTGTATCAT	630	Schmidt et al., 1995
		pCVD432/stop	CAATGTATAGAAATCCGCTGTT		

[a] Primers used for sequencing

Table S8. Targets and primers associated with extraintestinal pathotypes of *E. coli*

Pathotype	Target	Primers	Nucleotide sequence (5´- 3´)	Size (bp)	Reference
ExPEC	*kpsM II*	KpsII f	GCGCATTTGCTGATACTGTTG	272	Johnson & Stell, 2000
		KpsII r	CATCCAGACGATAAGCATGAGCA		
ExPEC	*kpsM II-K2*	kpsII f	GCGCATTTGCTGATACTGTTG	570	Johnson & O'Bryan, 2004
		KpsII-K2r	AGGTAGTTCAGACTCACACCT		
ExPEC	*kpsM II-K5*	K5 f	CAGTATCAGCAATCGTTCTGTA	159	Johnson & Stell, 2000
		kpsII r	CATCCAGACGATAAGCATGAGCA		
ExPEC	*neuC-K1*	neu1	AGGTGAAAAGCCTGGTAGTGTG	676	Moulin-Schouleur et al., 2006
		neu2	GGTGGTACATCCCGGGATGTC		
ExPEC	*kpsM III*	kps III f	TCCTCTTGCTACTATTCCCCCT	392	Johnson & Stell, 2000
		kps III r	AGGCGTATCCATCCCTCCTAAC		
ExPEC	*cvaC*	CoIV-Cf	CACACACAAACGGGAGCTGTT	680	Johnson & Stell, 2000
		CoIV-Cr	CTTCCCGCAGCATAGTTCCAT		
ExPEC	*ibeA*	Ibe10 f	AGGCAGGTGTGCGCCGCGTAC	170	Johnson & Stell, 2000
		Ibe10 r	TGGTGCTCCGGCAAACCATGC		
ExPEC	*iss*	is-f	CAGCAACCCGAACCACTTGATG	323	Johnson et al., 2008
		is-r	AGCATTGCCAGAGCGGCAGAA		
ExPEC	*malX*	MALX-F	GCATGAGCAGTGCGATACATCGC	828	Mora et al., 2013
		MALX-R	AGGGCTGGGAAGTGGTTTAGCC		
ExPEC	*traT*	TraTf	GGTGTGGTGCGATGAGCACAG	290	Johnson & Stell, 2000
		TraTr	CACGGTTCAGCCATCCCTGAG		
ExPEC	*tsh*	tsh03	GGTGGTGCACTGGAGTGG	640	Dozois et al., 2000
		tsh15	AGTCCAGCGTGATAGTGG		
ExPEC	*usp*	usp-f	ACATTCACGGCAAGCCTCAG	440	Bauer et al., 2002
		usp-r	AGCGAGTTCCTGGTGAAAGC		
ExPEC	*fimH*	FimH F	TGCAGAACGGATAAGCCGTGG	508	Johnson & Stell, 2000
		FimH R	GCAGTCACCTGCCCTCCGGTA		
ExPEC	*fimAv$_{MT78}$*	fimA215	ACTTTAGGATGAGTACTG	266	Marc & Dho-Moulin, 1996
		fimA201	TCTGGCTGATACTACACC		
ExPEC	*papC*	Forward	GTGGCAGTATGAGTAATGACCGTTA	205	Johnson et al., 2015
		Reverse	ATATCCTTTCTGCAGGGATGCAATA		
ExPEC	*sfa/focDE*	sfa 1	CTCCGGAGAACTGGGTGCATCTTAC	410	Le Bouguenec et al., 1992
		sfa 2	CGGAGGAGTAATTACAAACCTGGCA		
ExPEC	*afa/draBC*	afa1	GCTGGGCAGCAAACTGATAACTCTC	750	Le Bouguenec et al., 1992
		afa2	CATCAAGCTGTTTGTTCGTCCGCCG		
ExPEC	*cnf 1*	CNF1-F2	CAGGAGGTACTTAGCAGCGT	468	Mora et al., 2013
		CNF1-RC	TAATTTTGGGTTTGTATC		
ExPEC	*cdtB*	cdt-s1	GAAAGTAAATGGAATATAAATGTCCG	466	Tóth et al., 2003
		cdt-as1	AAATCTCCTGCAATCATCCAGTTA		
		cdt-s2	GAAAATAAATGGAACACACATGTCCG		
		cdt-as2	AAATCACCAAGAATCATCCAGTTA		
ExPEC	*hlyA*	hly f	AACAAGGATAAGCACTGTTCTGGCT	1177	Yamamoto et al., 1995
		hly r	ACCATATAAGCGGTCATTCCCGTCA		
ExPEC	*sat*	SatF	GCAGCTACCGCAATAGGAGGT	937	Johnson et al., 2003
		SatR	CATTCAGAGTACCGGGGCCTA		
ExPEC	*iucD*	Aer f	TACCGGATTGTCATATGCAGACCGT	602	Yamamoto et al., 1995
		Aer r	AATATCTTCCTCCAGTCCGGAGAAG		
ExPEC	*iro*N	Ironec-f	AAGTCAAAGCAGGGGTTGCCCG	665	Johnson et al., 2000
		Ironec-r	GACGCCGACATTAAGACGCAG		

Table S9. Detection and sequencing of *bla*$_{TEM}$, *bla*$_{SHV}$ and *bla*$_{CTX-M}$ genes

Target	Primers	Nucleotide sequence (5´- 3´)	Size (bp)	Reference
bla$_{CTX-M}$	CTX-C3	ATGTGCAGCACCAGTAAAGTGATG	542	Mora et al., 2013
	CTX-C4	ACCGCGATATCGTTGGTGGTGCC		
bla$_{CTX-M-grupo 1}$	M13U	GGTTAAAAAATCACTGCGTC	863	Saladin et al., 2002
	M13L	TTGGTGACGATTTTAGCCGC		
bla$_{CTX-M-grupo 1}$	[a]CTX-15-F1	GAAGCTAATAAAAAACACACGTGG	1044-1123	Mora et al., 2013
	[a]CTX-15-R	GTATGCGCAAGCGCAGGTGG		
bla$_{SHV}$	SHV-F2	TTGTCGCTTCTTTACTCGCC	879	Mora et al., 2013
	SHV-R2	CCCGGCGATTTGCTGATTTCGC		
bla$_{SHV}$	[a]SHV-1	GGGTTATTCTTATTTGTCGC	930	Rasheed et al., 1997
	[a]SHV-2	TTAGCGTTGCCAGTGCTC		
bla$_{TEM}$	[b]TEM-1F	ATGAGTATTCAACATTTCCG	868	Rasheed et al., 1997
	[b]TEM-1R	CTGACAGTTACCAATGCTTA		

[a] Primers used for sequencing; [b] Primers used for amplification and sequencing

Figure S1. GrapeTree inferred using the NINJA NJ algorithm and based on the cgMLST V1 + HierCC V1 scheme from Enterobase

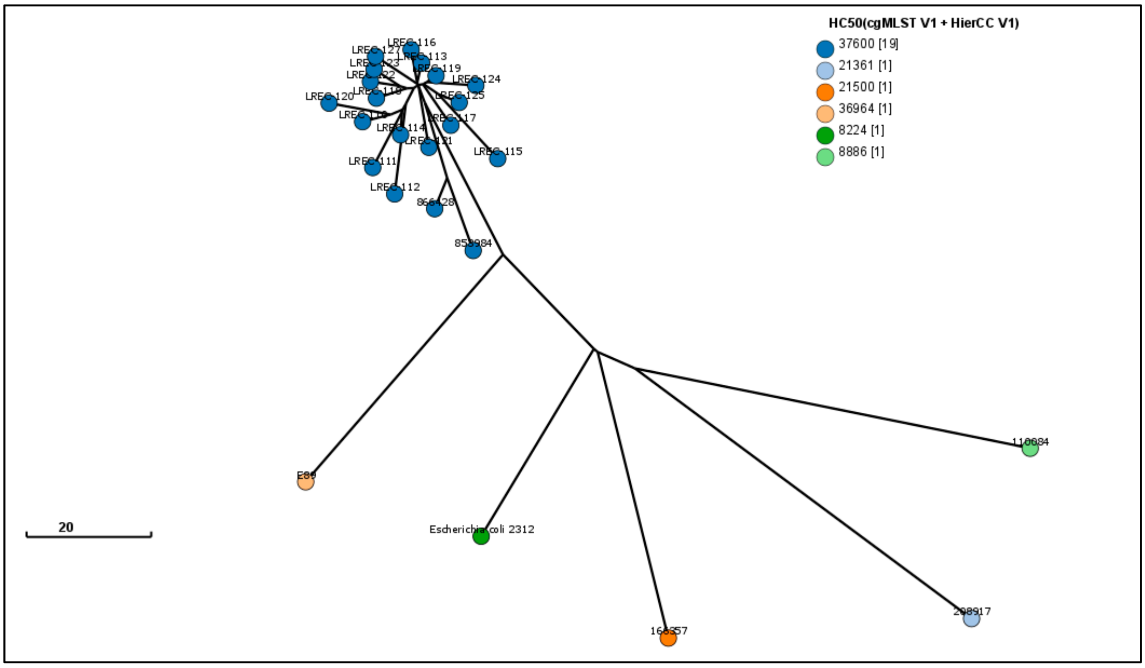

References

Alonso, C. A., Mora, A., Díaz, D., Blanco, M., González-Barrio, D., Ruiz-Fons, F., … Torres, C. (2017). Occurrence and characterization of stx and/or eae -positive Escherichia coli isolated from wildlife, including a typical EPEC strain from a wild boar. Veterinary Microbiology, 207, 69–73. https://doi.org/10.1016/j.vetmic.2017.05.028

Bauer, R. J., Zhang, L., Foxman, B., Siitonen, A., Jantunen, M. E., Saxen, H., & Marrs, C. F. (2002). Molecular Epidemiology of 3 Putative Virulence Genes for Escherichia coli Urinary Tract Infection— usp, iha, and iroN E. coli . The Journal of Infectious Diseases, 185(10), 1521–1524. https://doi.org/10.1086/340206

Bennett. (2003). Classical enteropathogenic Escherrichia coli or atypical strains? Examination of shigatoxin negative, eaeA positive isolates received in the Enteric Reference Laboratory in 2000. New Zealand Journal of Medical Laboratory Science, 57(1):2-7.

Blanco, M., Blanco, J. E., Gonzalez, E. A., Mora, A., Jansen, W., Gomes, T. A., … Blanco, J. (1997). Genes coding for enterotoxins and verotoxins in porcine Escherichia coli strains belonging to different O:K:H serotypes: relationship with toxic phenotypes. Journal of Clinical Microbiology, 35(11), 2958–2963. Retrieved from http://www.ncbi.nlm.nih.gov/pubmed/9350767

Dozois, C. M., Dho-Moulin, M., Brée, A., Fairbrother, J. M., Desautels, C., & Curtiss, R. (2000). Relationship between the Tsh autotransporter and pathogenicity of avian Escherichia coli and localization and analysis of the Tsh genetic region. Infection and Immunity, 68(7), 4145–4154. Retrieved from http://www.ncbi.nlm.nih.gov/pubmed/10858231

García-Meniño, I., García, V., Mora, A., Díaz-Jiménez, D., Flament-Simon, S. C., Alonso, M. P., … Blanco, J. (2018). Swine Enteric Colibacillosis in Spain: Pathogenic Potential of mcr-1 ST10 and ST131 E. coli Isolates. Frontiers in Microbiology, 9, 2659. https://doi.org/10.3389/fmicb.2018.02659

Johnson, J. R., Gajewski, A., Lesse, A. J., & Russo, T. A. (2003). Extraintestinal pathogenic Escherichia coli as a cause of invasive nonurinary infections. Journal of Clinical Microbiology, 41(12), 5798–5802. Retrieved from http://www.ncbi.nlm.nih.gov/pubmed/14662987

Johnson, J. R., & O'Bryan, T. T. (2004). Detection of the Escherichia coli group 2 polysaccharide capsule synthesis Gene kpsM by a rapid and specific PCR-based assay. Journal of Clinical Microbiology, 42(4), 1773–1776. Retrieved from http://www.ncbi.nlm.nih.gov/pubmed/15071046

Johnson, J. R., Porter, S., Johnston, B., Kuskowski, M. A., Spurbeck, R. R., Mobley, H. L. T., & Williamson, D. A. (2015). Host Characteristics and Bacterial Traits Predict Experimental Virulence for Escherichia coli Bloodstream Isolates From Patients With Urosepsis. Open Forum Infectious Diseases, 2(3), ofv083. https://doi.org/10.1093/ofid/ofv083

Johnson, J. R., Russo, T. A., Tarr, P. I., Carlino, U., Bilge, S. S., Vary, J. C., & Stell, A. L. (2000). Molecular epidemiological and phylogenetic associations of two novel putative virulence genes, iha and iroN(E. coli), among Escherichia coli isolates from patients with urosepsis. Infection and Immunity, 68(5), 3040–3047. Retrieved from http://www.ncbi.nlm.nih.gov/pubmed/10769012

Johnson, J. R., & Stell, A. L. (2000). Extended virulence genotypes of Escherichia coli strains from patients with urosepsis in relation to phylogeny and host compromise. The Journal of Infectious Diseases, 181(1), 261–272. https://doi.org/10.1086/315217

Johnson, T. J., Wannemuehler, Y. M., & Nolan, L. K. (2008). Evolution of the iss Gene in Escherichia coli. Applied and Environmental Microbiology, 74(8), 2360–2369. https://doi.org/10.1128/AEM.02634-07

Le Bouguenec, C., Archambaud, M., & Labigne, A. (1992). Rapid and specific detection of the pap, afa, and sfa adhesin-encoding operons in uropathogenic Escherichia coli strains by polymerase chain reaction. Journal of Clinical Microbiology, 30(5), 1189–1193. Retrieved from http://www.ncbi.nlm.nih.gov/pubmed/1349900

Marc, D., & Dho-Moulin, M. (1996). Analysis of the fim cluster of an avian O2 strain of Escherichia coli: serogroup-specific sites within fimA and nucleotide sequence of fimI. Journal of Medical Microbiology, 44(6), 444–452. https://doi.org/10.1099/00222615-44-6-444

Mora, A., Herrrera, A., López, C., Dahbi, G., Mamani, R., Pita, J. M., … Blanco, J. (2011). Characteristics of the Shiga-toxin-producing enteroaggregative Escherichia coli O104:H4 German outbreak strain and of STEC strains isolated in Spain. International Microbiology : The Official Journal of the Spanish Society for Microbiology, 14(3), 121–141. https://doi.org/10.2436/20.1501.01.142

Mora, A., Viso, S., López, C., Alonso, M. P., García-Garrote, F., Dabhi, G., … Blanco, J. (2013). Poultry as reservoir for extraintestinal pathogenic Escherichia coli O45:K1:H7-B2-ST95 in humans. Veterinary Microbiology, 167(3–4), 506–512. https://doi.org/10.1016/j.vetmic.2013.08.007

Moulin-Schouleur, M., Schouler, C., Tailliez, P., Kao, M.-R., Brée, A., Germon, P., … Blanco, J. (2006). Common virulence factors and genetic relationships between O18:K1:H7 Escherichia coli isolates of human and avian origin. Journal of Clinical Microbiology, 44(10), 3484–3492. https://doi.org/10.1128/JCM.00548-06

Penteado, A. S., Ugrinovich, L. A., Blanco, J., Blanco, M., Blanco, J. E., Mora, A., … Pestana de Castro, A. F. (2002). Serobiotypes and virulence genes of Escherichia coli strains isolated from diarrheic and healthy rabbits in Brazil. Veterinary Microbiology, 89(1), 41–51. Retrieved from http://www.ncbi.nlm.nih.gov/pubmed/12223161

Rasheed, J. K., Jay, C., Metchock, B., Berkowitz, F., Weigel, L., Crellin, J., … Tenover, F. C. (1997). Evolution of extended-spectrum beta-lactam resistance (SHV-8) in a strain of Escherichia coli during multiple episodes of bacteremia. Antimicrobial Agents and Chemotherapy, 41(3), 647–653. Retrieved from http://www.ncbi.nlm.nih.gov/pubmed/9056008

Saladin, M., Cao, V. T. B., Lambert, T., Donay, J.-L., Herrmann, J.-L., Ould-Hocine, Z., … Arlet, G. (2002). Diversity of CTX-M beta-lactamases and their promoter regions from Enterobacteriaceae isolated in three Parisian hospitals. FEMS Microbiology Letters, 209(2), 161–168. https://doi.org/10.1111/j.1574-6968.2002.tb11126.x

Schmidt, H., Knop, C., Franke, S., Aleksic, S., Heesemann, J., & Karch, H. (1995). Development of PCR for screening of enteroaggregative Escherichia coli. Journal of Clinical Microbiology, 33(3), 701–705. Retrieved from http://www.ncbi.nlm.nih.gov/pubmed/7751380

Schultsz, C., Pool, G. J., van Ketel, R., de Wever, B., Speelman, P., & Dankert, J. (1994). Detection of enterotoxigenic Escherichia coli in stool samples by using nonradioactively labeled oligonucleotide DNA probes and PCR. Journal of Clinical Microbiology, 32(10), 2393–2397. Retrieved from http://www.ncbi.nlm.nih.gov/pubmed/7814472

Tornieporth, N. G., John, J., Salgado, K., de Jesus, P., Latham, E., Melo, M. C., … Riley, L. W. (1995). Differentiation of pathogenic Escherichia coli strains in Brazilian children by PCR.

Journal of Clinical Microbiology, 33(5), 1371–1374. Retrieved from http://www.ncbi.nlm.nih.gov/pubmed/7615758

Tóth, I., Hérault, F., Beutin, L., & Oswald, E. (2003). Production of cytolethal distending toxins by pathogenic Escherichia coli strains isolated from human and animal sources: establishment of the existence of a new cdt variant (Type IV). Journal of Clinical Microbiology, 41(9), 4285–4291. Retrieved from http://www.ncbi.nlm.nih.gov/pubmed/12958258

Yamamoto, S., Terai, A., Yuri, K., Kurazono, H., Takeda, Y., & Yoshida, O. (1995). Detection of urovirulence factors in Escherichia coli by multiplex polymerase chain reaction. FEMS Immunology and Medical Microbiology, 12(2), 85–90. https://doi.org/10.1111/j.1574-695X.1995.tb00179.
